# The dynamic alteration of transcriptional regulation by crucial TFs during tumorigenesis of gastric cancer

**DOI:** 10.1186/s10020-022-00468-7

**Published:** 2022-04-14

**Authors:** Beiqin Yu, Wentao Dai, Li Pang, Qingqing Sang, Fangyuan Li, Junxian Yu, Haoran Feng, Jianfang Li, Junyi Hou, Chao Yan, Liping Su, Zhenggang Zhu, Yuan-Yuan Li, Bingya Liu

**Affiliations:** 1 Department of General Surgery, Shanghai Key Laboratory of Gastric Neoplasms, Shanghai Institute of Digestive Surgery, Ruijin Hospital, Shanghai Jiao Tong University School of Medicine, Shanghai, 200025 China; 2 NHC Key Lab of Reproduction Regulation (Shanghai Institute for Biomedical and Pharmaceutical Technologies), Fudan University, Shanghai, 200080 China; 3 Shanghai Engineering Research Center of Pharmaceutical Translation, Shanghai, 201203 China; 4 Department of Surgery, HKU-SZH and Faculty of Medicine, The University of Hong Kong, Hong Kong, China

**Keywords:** Gastric cancer, Differential co-expression analysis, Gene regulatory network, Differential regulation analysis, Transcription factors

## Abstract

**Background:**

The mechanisms of Gastric cancer (GC) initiation and progression are complicated, at least partly owing to the dynamic changes of gene regulation during carcinogenesis. Thus, investigations on the changes in regulatory networks can improve the understanding of cancer development and provide novel insights into the molecular mechanisms of cancer.

**Methods:**

Differential co-expression analysis (DCEA), differential gene regulation network (GRN) modeling and differential regulation analysis (DRA) were integrated to detect differential transcriptional regulation events between gastric normal mucosa and cancer samples based on GSE54129 dataset. Cytological experiments and IHC staining assays were used to validate the dynamic changes of CREB1 regulated targets in different stages.

**Results:**

A total of 1955 differentially regulated genes (DRGs) were identified and prioritized in a quantitative way. Among the top 1% DRGs, 14 out of 19 genes have been reported to be GC relevant. The four transcription factors (TFs) among the top 1% DRGs, including CREB1, BPTF, GATA6 and CEBPA, were regarded as crucial TFs relevant to GC progression. The differentially regulated links (DRLs) around the four crucial TFs were then prioritized to generate testable hypotheses on the differential regulation mechanisms of gastric carcinogenesis. To validate the dynamic alterations of gene regulation patterns of crucial TFs during GC progression, we took CREB1 as an example to screen its differentially regulated targets by using cytological and IHC staining assays. Eventually, TCEAL2 and MBNL1 were proved to be differentially regulated by CREB1 during tumorigenesis of gastric cancer.

**Conclusions:**

By combining differential networking information and molecular cell experiments verification, testable hypotheses on the regulation mechanisms of GC around the core TFs and their top ranked DRLs were generated. Since TCEAL2 and MBNL1 have been reported to be potential therapeutic targets in SCLC and breast cancer respectively, their translation values in GC are worthy of further investigation.

**Supplementary Information:**

The online version contains supplementary material available at 10.1186/s10020-022-00468-7.

## Background

Gastric cancer (GC) remains one of the most common malignant tumors and a leading cause of cancer mortality worldwide, especially in East Asia (Chen et al. 2016; Siegel et al. 2019). Targeted therapy has shown limited efficacy in GC patients, since HER2-targeted Trastuzumab, VEGFA/VEGFR2-targeted Bevacizumab, Ramucirumab, Apatinob, Regorafenib, and some immunomodulators such as Nivolumab for PD-L1 positive metastatic GC, are the only effective therapies so far, and most clinical trials evaluating targeted treatments with approved efficacy in other cancer types have failed in gastric cancer. Studies on molecular mechanisms would help to explore novel targets and therapies. In addition to the efforts aiming to highlight molecular alterations in potential driver genes, it is necessary to clarify the dynamic alteration of transcriptional regulation during the process of gastric carcinogenesis.

Transcription factors (TFs), being the hubs of gene regulatory network (GRN) and cellular signaling, participate in transcription regulation during growth and development of both normal and tumor tissues. It has been widely accepted that transcriptional dysregulation plays a key role in carcinogenesis, metastasis, prognosis and drug dependency in a large number of cancers. Therefore, deciphering conditional regulation patterns from dynamic GRNs and elucidating the mechanisms of gene dysregulation triggered by oncogenic TFs is critical for understanding the molecular biology of cancer and designing effective therapeutic strategies (Gascard et al. 2015; Thoms et al. 2019). In this scenario, a realistic problem is how to find the key TFs with distinct regulation functions in normal and cancer tissues, and further how to understand the roles of these TFs in different states.

In recent years, differential co-expression analysis (DCEA) is emerging as a practical approach that focuses on the changes in gene co-expression patterns between two phenotypes rather than the traditional focus, i.e., the changes in expression level of individual genes, and thus provides clues to the abnormal regulations specific to the phenotype of interest (de la Fuente, 2010; Yu et al. 2011). Furthermore, gene regulation network modeling (GRN modeling) and differential regulation analysis (DRA) enables the identification of differential regulatory relationships during phenotypic changes or pathological processes (Hood et al. 2004). In the present study, by integrating DCEA, GRN modeling and DRA, we obtained four differentially regulated TFs, CREB1, BPTF, GATA6 and CEBPA, and their surrounding differentially regulated links (DRLs), which provided novel insights into the pathophysiology of GC carcinogenesis. Further experimental verification indicated that CREB1 might contribute to GC progression by differentially regulating TCEAL2 and MBNL1.

## Methods

### Cell culture

GC cell lines NCI-N87 and BGC823 were obtained from Shanghai Institutes for Biological Sciences, Chinese Academy of Sciences. An immortalized normal gastric epithelial cell line GES-1 and 293 T cells were cultured in RPMI-1640 or DMEM medium supplemented with 10% fetal calf serum and maintained at 37 °C in a humidified atmosphere of 5% CO_2_.

### Tissues

GC tissues and normal tissues were obtained from patients who underwent radical gastrectomy between 2013 and 2016 at the Department of Surgery, Ruijin Hospital, Shanghai Jiao Tong University School of Medicine. All tissues including 56 GC samples and 52 normal samples were formalin-fixed and paraffin-embedded into tissue arrays. All samples were confirmed by pathological diagnosis.

### Gene expression profiles

The gene expression profile dataset GSE54129 (111 gastric cancer and 21 normal mucosa samples) were customized using Human Whole Genome U133 Plus 2.0 array (Affymetrix Inc, Santa Clara, CA, USA). Microarray quality control and assessment were performed using R affy package (Gautier et al. 2004) available from the Bioconductor website. The expression data were normalized by RMA (robust multi-array average) method and log2 transformed. The data quality was estimated according to express level distribution, density distribution and correlations of samples.

### Differential co-expression and differential expression analysis

Differentially co-expressed genes (DCGs) and differentially co-expressed gene links (DCLs) were obtained with our differential co-expression analysis (DCEA) algorithms (Liu et al. 2010; Yang et al. 2013; Yu et al. 2011). Specifically, DCGs were identified by DCp method with FDR less than 0.05 and DCLs were identified by modified LFC method. Differentially expressed genes (DEGs) were obtained by SAM method (Dudoit et al. 2002) with q-value less than 0.05. The expression profile of DEGs in GSE54129 was clustered by using ward linkage hierarchical clustering method (Murtagh and Legendre 2014).

### Differential regulation network modeling and analysis

Following the protocol described in our previous work (Cao et al. 2015), we constructed stage-specific GRNs by using multivariant linear regression model based on the expression profile of DCGs identified from GSE54129 and candidate TF-target regulatory relationships from UCSC (http://genome.ucsc.edu/). Through prefiltering regulators and stepwise linear regression, mRNA expression level of the DCGs in GSE54129 was modeled by its crucial regulators with quantitative regulation efficacies in these stage-specific GRNs.

To identify the differential regulation gene (DRG) in GRNs, a quantitative differential regulation (DR) measure was adopted to capture the average regulation changes of a gene between two GRNs, ${DR}_{i}=\sqrt{\frac{{\sum }_{j=1}^{n}{({X}_{ij}-{Y}_{ij})}^{2}}{n}}$, with X_ij_ and Y_ij_ as the regulation efficacies between gene i and j in GRN X and Y, respectively (Cao et al. 2015). In order to identify and measure differentially regulated links (DRLs) between two GRNs, the method combined with modified LFC method and regulatory efficiency log fold change curve filter was used (Cao et al. 2015; Liu et al. 2010).

### CREB1 knockdown and overexpression

For CREB1 knockdown, a CREB1-siRNA sequence 5′-CCAACAAAUGACAGUUCAATT-3′ and the negative control sequence 5′-GTTCTCCGAACGTGTCACGT-3′ were synthesized by GenePharma (Shanghai, China). Transfection of cells with oligonucleotides was performed using Lipofectamine 2000 Reagent (Invitrogen, Carlsbad, CA, USA) at a final concentration of 100 nM. Transfection efficiency was monitored by qRT-PCR and Western blot. The Ubi-MCS-SV40/CREB1 lentiviral transduction particles for ectopic overexpression of CREB1 was purchased from Genechem (Shanghai, China) and transfected into cells. Stable transfected cell clones were selected with puromycin and screened by qRT-PCR and Western blot.

### Quantitative RT-PCR (qRT-PCR)

QRT-PCR was performed as described before (Yu et al. 2017). Each assay was repeated 3–4 times. The primer sequences for in vitro assays are available in Additional file 8: Table S1.

### Western blot

Western blot was performed as described before (Yu et al. 2017). The primary antibodies against CREB1 (1:1000, Abcam), TCAEL2 (1:1000, Abcam) and MBNL1 (1:1000, Abcam) were used. Goat anti-mouse or goat anti-rabbit IgG conjugated with horseradish peroxidase (HRP, ProteinTech) dilutions were 1:10,000. Primary antibody against GAPDH from ProteinTech was used as a control to confirm equal loading of proteins.

### Immunohistochemistry staining (IHC)

IHC staining was performed as previously reported (Yu et al. 2017). Four duplicate tissue arrays containing 56 GC samples and 52 normal samples were incubated by primary antibodies including anti-CREB1 (1:500, Abcam), MBNL1 (1:250, Abcam) and anti-TCAEL2 (1:250, Abcam). The percentage of positive cells was divided into five grades (percentage scores): < 5% (0), 5–25% (1), 25–50% (2), 50–75% (3), 75–100% (4). The intensity of staining was divided into four grades (intensity scores): no staining (0), weak staining (1), moderate staining (2) and strong staining (3). The final IHC staining score was determined by the following formula: overall score = percentage score × intensity score. The overall score ≤ 3 was defined as negative, and > 3 as positive.

### Luciferase reporter assay

TCEAL2 or MBNL1 promoter fragments were amplified from human genomic DNA, and were inserted into pGL3-Basic vector. Luciferase activity was measured after 24 h incubation using a Dual-Glo luciferase assay kit (Promega) and single-tube luminometer (Promega).

### Statistical analysis

Results were shown as mean ± standard deviation (SD). The expression correlations between CREB1 and its targets were analyzed by Spearman test. Differences between experimental groups were assessed by the Student’s t test or one-way ANOVA. A result with two-tailed p-value < 0.05 was deemed as statistically significant. Statistical analyses were performed using SPSS 22.0 software (SPSS Inc.).

## Results

### Stage-specific gene regulatory networks

The gene expression profile of GC (GSE54129) involves 111 gastric cancer and 21 normal mucosa samples (see Additional file 1: Fig. S1A–C for data quality assessment). By using R affy package, 2415 DEGs were obtained with FDR < 0.05 and log2 fold change > 1.5. Unsupervised hierarchical clustering based on the 2415 DEGs out of the 132 samples exhibited a clear separation between normal and cancer, indicating the reliability of the data and the clinical relevance of the differential expression between cancer and normal (Additional file 1: Fig. S1D).

**Fig. 1:**
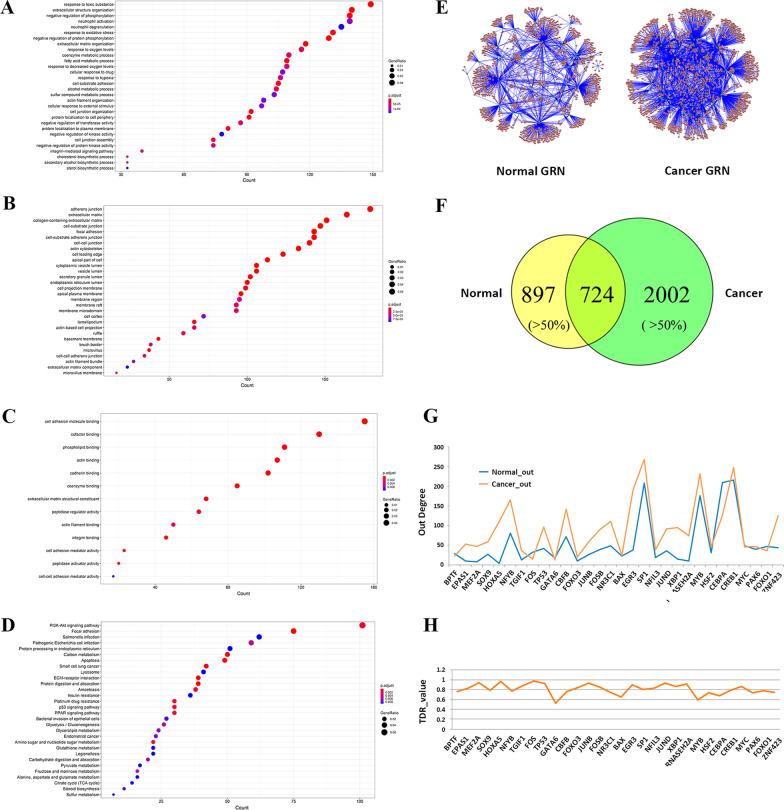
Stage-specific gene regulatory networks and GO analysis. GO analysis of the DCGs in the terms of (**A**) Biological processes; (**B**) Cellular components; (**C**) Molecular function and (**D**) KEGG pathway analysis. **E** Gene regulatory network (GRN) in normal (left) and in cancer (right). **F** Venn diagrams illustrate the number of regulatory relationships involved in two stage-specific GRNs. **G** Out-degree distribution of 30 TFs involved in two stage-specific GRNs. **H** Distribution of TDR values of 30 TFs in two stage-specific GRNs

We then applied DCGL package to GSE54129 dataset and extracted a total of 3875 DCGs between normal and cancer. In order to confirm the functional relevance of the DCGs, we carried out functional enrichment analysis in Gene Ontology by using DAVID (https://david.ncifcrf.gov/home.jsp) and obtained a number of biological processes, including response to toxic substance, extracellular structure organization and negative regulation of phosphorylation and so on (Fig. 1A), a number of cellular components, including adherens junction, extracellular matrix, collagen-containing extracellular matrix and so on (Fig. 1B), and several molecular functions, including cell adhesion molecule binding, cofactor binding, phospholipid binding and so on (Fig. 1C). In addition, these DCGs were also enriched in three KEGG pathways, PI3K-Akt signaling pathway, Focal adhesion and Salmonella infection (Fig. 1D). These observations are coherent to our general understanding of cancer progression.

Based on the stage-specific expression data of the DCGs, stage-specific GRNs corresponding to normal and cancer were separately constructed by using stepwise linear regression method (Fig. 1E). Aiming to investigate the dynamic changes of gene regulation during GC progression, the topological properties of the two GRNs were compared in terms of their basic statistics (Table 1) and topological parameters including in-degree (In-Deg), out-degree (Out-Deg), betweenness (Bet), clustering coefficient (CC), and closeness (Cls) (Table 2). First of all, the regulatory relationships (links) of cancer network (2726) were about 70.3% more than the normal network (1621) (Table 1). Accordingly, the In-Deg, CC and Cls are significantly different between normal and cancer networks according to Wilcoxon rank-sum test (Table 2), indicating that the topological structure of gene regulation networks greatly changed to be more compact during GC progression. When classifying the links into “overlapped” and “stage-specific”, the number of stage-specific links accounted for over 50% for both two networks (Fig. 1F), supporting that our stage-specific GRNs were indeed enriched with differential regulatory relationships. It was interesting that the two GRNs share the same 30 TFs (Table 1), however, the regulation patterns around these TFs were apparently different between normal and cancer stages. Specifically, although the Out-Deg values of the 30 TFs showed a similar pattern between two GRNs (Fig. 1G), more than 50% targets regulated by a certain TF changed between two GRNs according to the TDR (target diversity of a regulator) measure (Fig. 1H) which was used to quantitatively examine how the TF targets changed across GRNs(Cao et al. 2015). Therefore, we propose the 30 TFs involved in our GRNs to be relevant to differential regulation underlying gastric carcinogenesis.

**Table 1:** Statistics of stage-specific GRNs in GSE54129

GRNs	Links	TFs	Targets
Normal	1621	30	1242
Cancer	2726	30	1657

**Table 2:** Toplogical comparison of stage-specific GRNs

	In-Deg	Out-Deg	Bet	CC	Cls
Normal	1.31	54.08	0.12	0.045	6.32E-07
Cancer	1.64	90.89	2.46	0.09	3.66E-07
Normal vs*.* Cancer	3.39E−44*	0.281	0.509	3.61E−06*	0*

^*^Means significant p-value < 0.05. Topological difference significance (p-value) was calculated by Wilcoxon rank-sum test

### Differentially regulated genes (DRGs) between normal and tumor tissues

We applied differential regulation analysis (DRA) to capture differential regulation events during phenotypic changes (Cao et al. 2015), and obtained a total of 1955 DRGs between normal and cancer GRNs. (see Additional file 9: Table S2 for the DRG list in a descending order by DR value). According to the design of DR measure, DRGs have experienced significant change during phenotypic progression, and the change could be related to TF-target relationships and the regulation efficacy. We propose that genes with higher ranks in the DRG list undergo more significant functional alteration and play more important roles in gastric cancer progression. As expected, fourteen out of the top 1% (nineteen) DRGs (Table 3) have been reported to be GC relevant (BPTF (Lee et al. 2016), REG4 (Duan et al. 2014), ANXA13 (van Duin et al. 2007), C7 (Tsuge et al. 2009), ASS1 (Tsai et al. 2018), MSMB (Ohnuma et al. 2009), CREB1 (Liu et al. 2019), GATA6 (Song et al. 2018), TRIM15 (Zhou et al. 2020), GAST (Tang et al. 2019), SLC39A5 (Ding et al. 2019), CDCA3 (Yu et al. 2019), CPS1 (Hejna et al. 2016) and CEBPA (Shi et al. 2015)). We then took the four TFs: CREB1, BPTF, GATA6 and CEBPA among the top 1% ranked DRGs as crucial TFs relevant to GC progression.

**Table 3:** The top 1% genes in DRG list

DRGs	DR_value	Rank
SLC7A9	65.03438	1
**REG4**	22.26006	2
***BPTF***	20.02962	3
DHRS11	19.11607	4
**ANXA13**	17.92791	5
**C7**	15.36403	6
**ASS1**	15.10191	7
**MSMB**	13.03145	8
CLCA1	12.91625	9
***CREB1***	12.84939	10
***GATA6***	10.11172	11
**TRIM15**	9.481667	12
CLRN3	9.299721	13
**GAST**	8.957028	14
DHRS1	8.936077	15
**SLC39A5**	8.710737	16
**CDCA3**	8.526506	17
**CPS1**	7.973394	18
***CEBPA***	7.791725	19

The genes are sorted by the DR values. Genes in bold refer to

GC-related genes; genes in italic refer to TFs

### Differentially regulated links (DRLs) around CREB1, BPTF, GATA6 and CEBPA

In order to understand the dynamic changes in regulation relationships of crucial TFs, we investigated the differentially regulated links (DRLs) around CREB1, BPTF, GATA6 and CEBPA. DRLs around each TF across the two GRNs were obtained by using the modified LFC model from DCGL package (Cao et al. 2015; Liu et al. 2010), and ranked by their absolute changes of regulation efficacy in a descending order. Similarly as DRGs, we proposed that gene pairs with higher ranks in the DRL list play more important roles in gastric cancer progression.

**Fig. 2:**
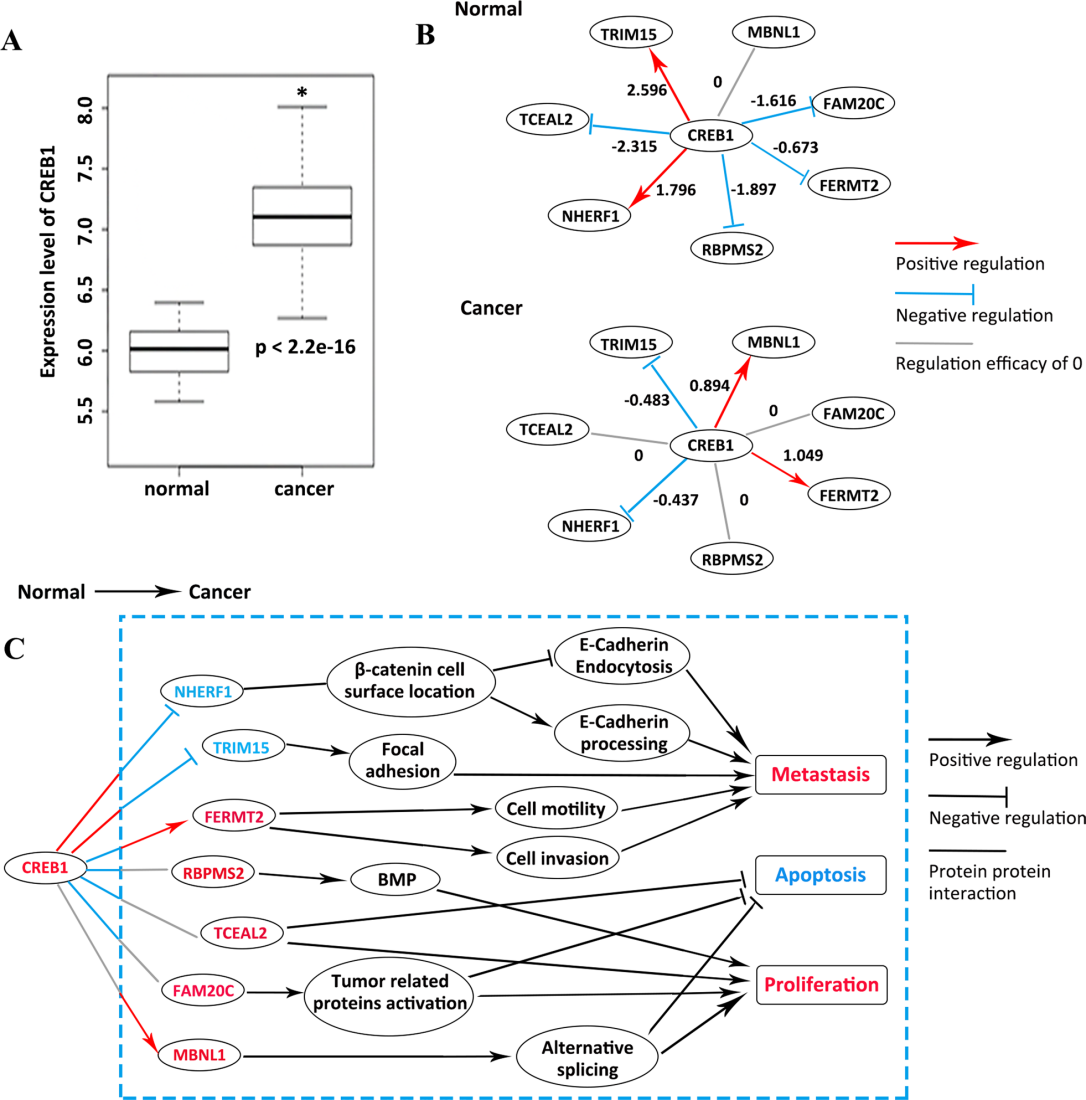
The proposed dysregulation mechanisms around CREB1. **A** The expression level of CREB1 in GSE54129 dataset. **B** CREB1 is a TF, and the other nodes are its targets. Links in red, blue and grey represent positive, negative and absent relationships calculated with dataset GSE54129. Numbers on the links indicate the regulation efficacies. **C** The proposed mechanism by which CREB1 induces GC. *Means significant *P*-value < 0.05, two-sided Student’s *t*-test

CREB1 is an oncogenic TF in GC and plays critical role in physiological processes (Siu and Jin 2007). The expression level of CREB1 was elevated in cancer samples in GSE54129 (Fig. 2A), which is consistent with the observation in STAD (http://gepia.cancer-pku.cn/detail.php?gene=CREB1). The transcriptional regulation activities of CREB1 were reported to impart selectivity across different conditions (Mayr et al. 2001). The top seven ranked differential DRLs (TRIM15, TCEAL2, NHERF1, RBPMS2, FERMT2, FAM20C and MBNL1) around CREB1 were ordered descendingly by the changes of regulation efficacy in Table 4. According to the regulation efficacy data (Table 4), the positive regulation of TRIM15 and NHERF1 by CREB1 was reversed from normal to cancer; the negative regulations of TCEAL2, RBPMS2 and FAM20C by CREB1 disappeared; and the negative regulation of FERMT2 was reversed from normal to cancer. MBNL1 was not regulated by CREB1 in normal, while positively regulated by CREB1 in cancer. The differential expression trends of the seven target genes in GSE54129 dataset are consistent with the above regulation alteration. The dysregulation events induced by CREB1 and their functional relevance were summarized in Fig. 2B, C. We proposed that CREB1 could differentially regulate its downstream targets between normal and cancer tissues, which at least partly leads to GC development.

**Table 4:** The regulation efficacy of CREB1 on its targets

TF	Target	Normal	Cancer	DRL_value
CREB1	TRIM15	2.596	− 0.483	3.079
CREB1	TCEAL2	− 2.315	0.000	2.315
CREB1	NHERF1	1.796	− 0.437	2.233
CREB1	RBPMS2	− 1.897	0.000	1.897
CREB1	FERMT2	− 0.673	1.049	1.722
CREB1	FAM20C	− 1.616	0.000	1.616
CREB1	MBNL1	0	0.894	0.894

BPTF plays a critical role in embryogenesis and stem cell differentiation and has been reported to participate in the initiation and progression of multiple tumors (Richart et al. 2016; Stankiewicz et al. 2017). According to our GSE54129 dataset, the expression level of BPTF was increased in gastric cancer (Additional file 2: Fig. S2A) and furthermore, BPTF formed DRLs with PPM1L, NKX6.3 and PIK3R3 (Additional file 2: Fig. S2B). The positive regulation of PPM1L by BPTF was lost from normal to cancer, and meanwhile the expression of PPM1L was found to be decreased in cancer, which is consistent with the regulation change (Thean et al. 2010). The positive regulation of NKX6.3 by BPTF was reversed from normal to cancer, and consistently, the decreased expression of NKX6.3 was observed in cancer. The negative regulation of PIK3R3 by BPTF was lost from normal to cancer, which was in accordance with the over-expression of PIK3R3 in cancer. Taken together, BPTF may exert its oncogenic role in GC through differentially regulating downstream targets PPM1L, WKX6.3 and PIK3R3 (Fig. S2C), which was supported by the established association with carcinogenesis of PPM1L (Thean et al. 2010), WKX6.3 (Yoon et al. 2016) and PIK3R3 (Yu et al. 2015).

GATA6, a critical regulator in the development of gastrointestinal tract, has been found to control apoptosis and cell cycle of GC. Previous study reported that GATA6 expression was decreased in GC and GATA6 may act as a tumor suppressor (Sulahian et al. 2014). We also observed decreased expression of GATA6 in cancer (Additional file 3: Fig. S3A). Additionally, GATA6 formed DRLs with REG4, CA9, and STC1 in our stage-specific GRNs (Additional file 3: Fig. S3B). The negative regulation of REG4 by GATA6 was reversed to be positive from normal to cancer, the positive regulation of CA9 by GATA6 in normal tissue disappeared in cancer and the negative regulation of STC1 by GATA6 in normal stage was also disappeared in cancer. Similarly, the changes in gene expression of the three targets are consistent with the change in transcriptional regulation. Combined with prior knowledge from literature, the dysregulation mechanisms around GATA6 were proposed in Additional file 3: Fig. S3C.

CEBPA acts a crucial role in terminal differentiation, and has been proved to be a tumor suppressor gene in GC (Altarejos and Montminy 2011). The expression of CEBPA was decreased in cancer samples in GSE54129 dataset (Additional file 4: Fig. S4A) and formed DRLs with CLCA1, CES2 (Additional file 4: Fig. S4B). The positive regulation of CLCA1 and CES2 were both lost from normal to cancer, and thus may result in reduced expression of two targets, which could further inhibit proliferation or induce apoptosis (Li et al. 2017b; Shaojun et al. 2018) (Additional file 4: Fig. S4C).

### Differentially regulated targets of CREB1 between normal and cancer

**Fig. 3:**
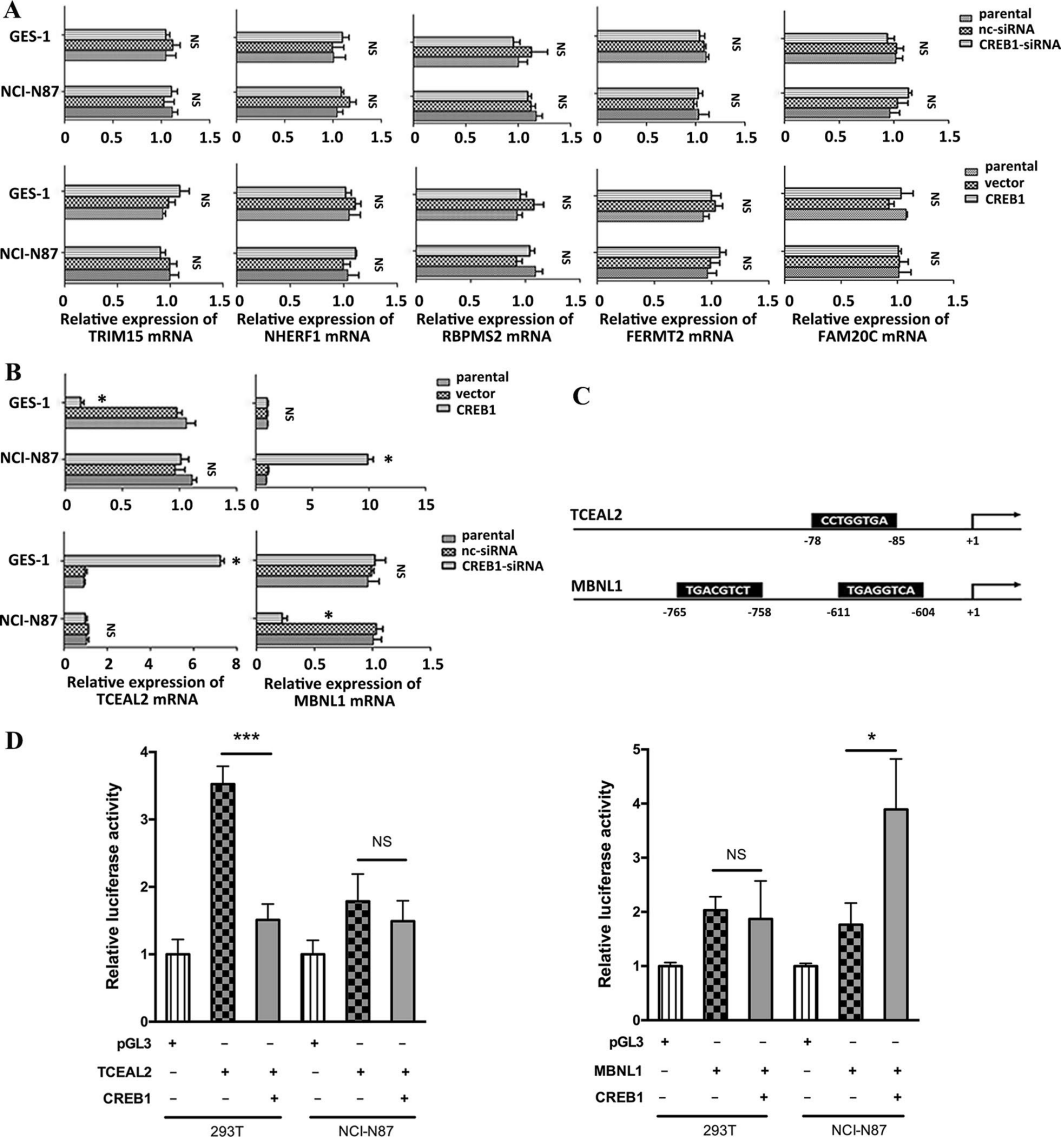
Validation of differentially regulated targets of CREB1 between normal and cancer. **A** TRIM15, NHERF1, RBPMS2, FERMT2 and FAM20C, each gene expression was measured by qRT-PCR in GES-1 and NCI-N87 cells transfected with CREB1 specific siRNA or CREB1 lentivirus (n = 3–4). **B** Overexpression or downregulation of CREB1 in GES-1 and NCI-N87 cells, and the expression changes of TCEAL2 and MBNL1 were measured by qRT-PCR (n = 3–4). **C** CREs in the promoters of TCEAL2 and MBNL1. Sequence analysis of the promoters of the two genes indicated the potential binding sites for CREB1. **D** TCEAL2 or MBNL1 pGL3 luciferase reporter was co-transfected with CREB1 into 293 T cells and NCI-N87 cells, and relative luciferase activity was detected. *Means significant *P*-value < 0.05, *** means significant *P*-value < 0.001, NS means no significant, two-sided Student’s *t*-test

In order to confirm the effects of differential regulation by CREB1, we examined the changes in mRNA expression of the seven candidate dysregulated targets of CREB1, including TRIM15, TCEAL2, NHERF1, RBPMS2, FERMT2, FAM20C and MBNL1, after silencing and overexpressing CREB1 in immortalized gastric epithelial cell line GES-1 and GC cell line NCI-N87, respectively. TRIM15, NHERF1, RBPMS2, FERMT2 and FAM20C did not show any significant expression change after disturbing CREB1 expression in both normal gastric epithelial cells and GC cells, and therefore were filtered out from the target list (Fig. 3A). Consistent with the modeling result that the negative regulation of TCEAL2 by CREB1 disappeared from normal to cancer, the expression of TCEAL2 was markedly decreased in GES-1/CREB1 and increased in GES-1/CREB1-siRNA compared with control group, while the expression of TCEAL2 in NCI-N87 kept stable between disturbed (CREB1 or CREB1-siRNA) and control (Fig. 3B). In accordance with the modeling result that MBNL1 was only positively regulated by CREB1 in cancer, the expression of MBNL1 in GES-1 did not show any significant change between disturbed (CREB1 or CREB1-siRNA) and control, while it was significantly increased in NCI-N87/CREB1 and decreased in NCI-N87/CREB1-siRNA compared with control (Fig. 3B). Similar results were obtained in BGC823 cells with CREB1 overexpression or knockdown (Additional file 5: Fig. S5A, B).

As shown in Fig. 3C, TCEAL2 and MBNL1 contain putative responsive elements (CREs) of CREB1 in their promoter regions according to JASPAR, CCTGGTGA and TGAC/GGTCT/A. To verify the transcriptional role of CREB1 in TCEAL2 or MBNL1 promoter, CREB1 plasmid was co-transfected with TCEAL2 or MBNL1 luciferase reporter plasmid containing CREB1 binding sites into 293 T cells and NCI-N87 cells. Renilla luciferase was applied as a control for normalization of transfection efficiency. It was found that CREB1 overexpression suppressed TCEAL2 promoter in 293 T cells and activated MBNL1 promoter in NCI-N87 cells (Fig. 3D).

### Differential regulation of TCEAL2 and MBNL1 by CREB1 between normal and cancer

**Fig. 4:**
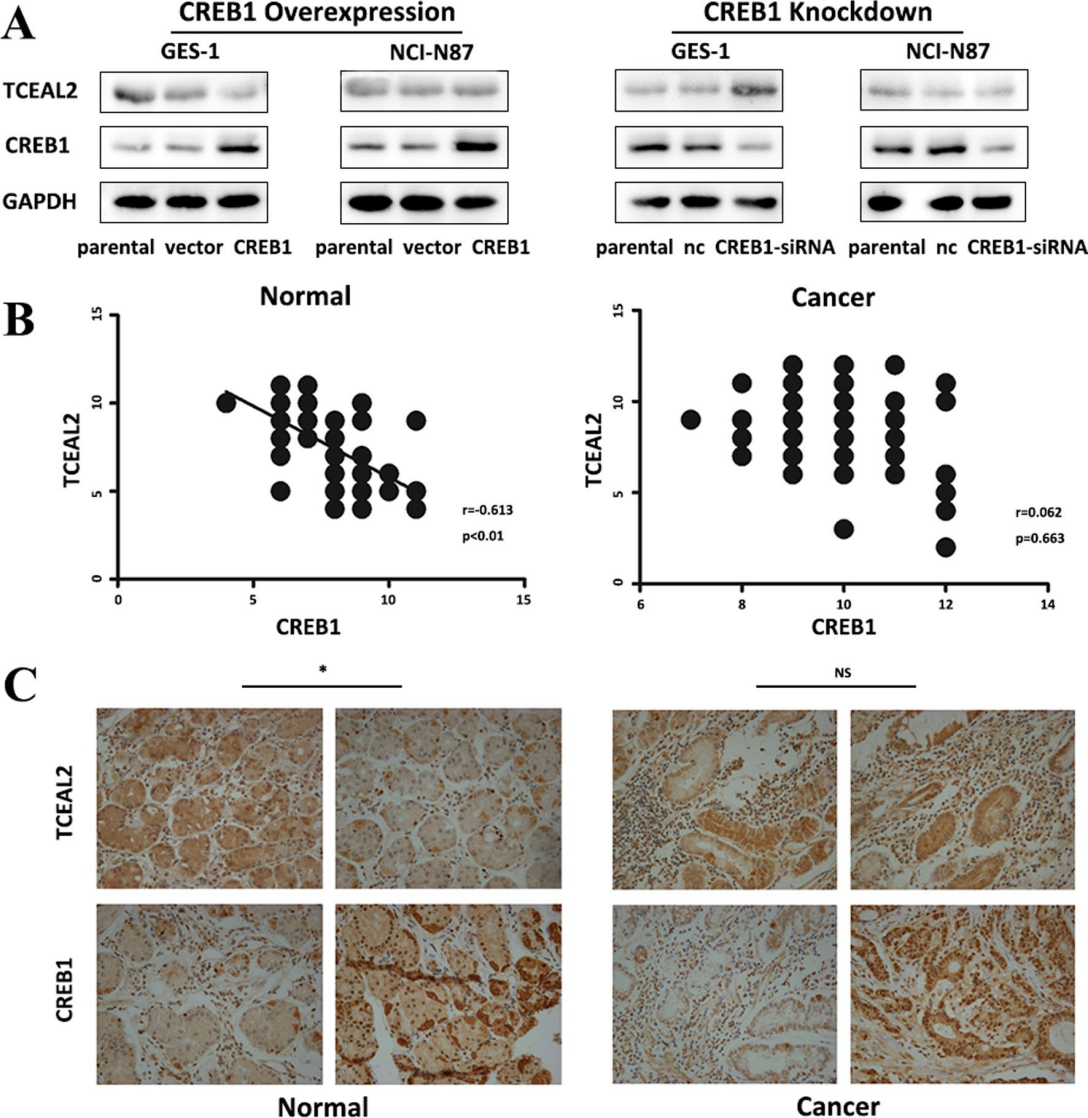
CREB1 differentially regulated TCEAL2 under normal and cancer stages.** A** Western blot analysis for TCEAL2 expression was performed with cell lysate from GES-1 and NCI-N87 transfected with CREB1 lentivirus vectors (Left) or siRNA specific to CREB1 (Right). **B** Correlation between CREB1 and TCEAL2 in diagnostic tumor samples (n = 56, *r* = 0.062, *P* = 0.663) and normal samples (n = 52, *r* = 0.613, *P* < 0.01). **C** Representative IHC staining of CREB1 and TCEAL2 in tumor and normal samples (400 ×). *Means significant *P*-value < 0.05, NS means no significant, correlation between CREB1 and TCAEL2 expression was analyzed by Spearman test

In order to confirm the effects of differential regulation of TCEAL2 and MBNL1 by CREB1 at the protein level, western blot and IHC were performed for each target. The protein level of TCEAL2 was increased in GES-1/siCREB1 and reduced in GES-1/CREB1 in comparison with control groups (Fig. 4A). However, in NCI-N87/siCREB1 and NCI-N87/CREB1, TCEAL2 didn’t show any differences compared with control groups (Fig. 4A). In BGC823 cells, TCEAL2 had no change after CREB1 disturbing either (Fig. S5C and S5D). IHC staining revealed that the expression of TCEAL2 was negatively correlated with CBRE1 in normal gastric samples (n = 52, *r* = − 0.613, *P* < 0.01), whereas no correlation with CREB1 in GC samples (n = 56, *r* = 0.062, *P* = 0.663) (Fig. 4B, C).

**Fig. 5:**
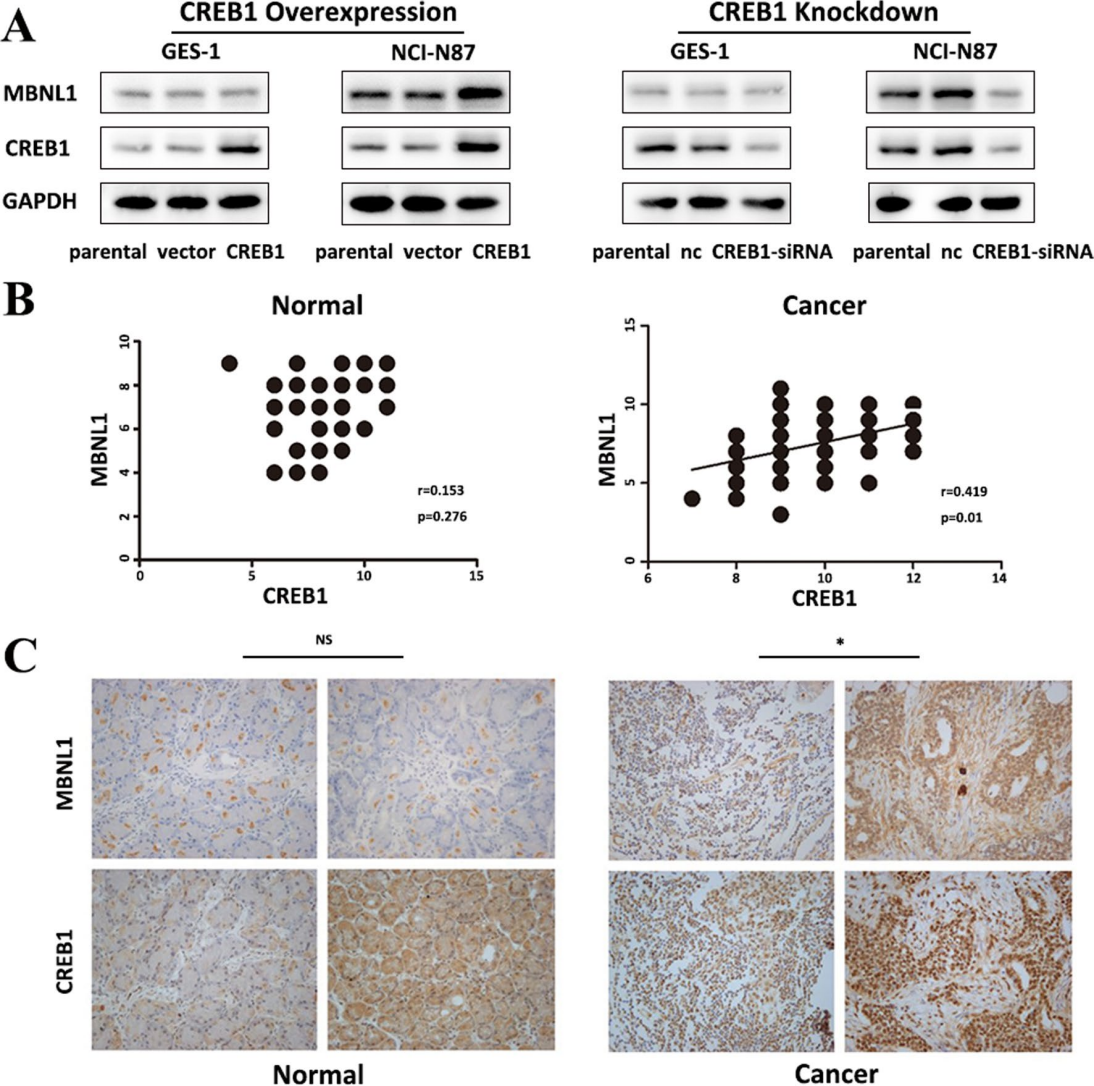
CREB1 differentially regulated MBNL1 under normal and cancer stages. **A** Western blot analysis for MBNL1 expression was performed with cell lysate from GES-1 and NCI-N87 transfected with CREB1 lentivirus vectors (Left) or siRNA specific to CREB1 (Right). **B** Correlation between CREB1 and MBNL1 in diagnostic tumor samples (n = 56, *r* = 0.419, *P* = 0.01) and normal samples (n = 52, *r* = 0.153, *P* = 0.276). **C** Representative IHC staining of CREB1 and MBNL1 in tumor and normal samples (400 ×). *Means significant *P*-value < 0.05, NS means no significant, correlation between CREB1 and MBNL1 expression was analyzed by Spearman test

MBNL1 was upregulated in NCI-N87/CREB1 and reduced in NCI-N87/siCREB1 compared with control groups, however, the expression of MBNL1 didn’t change obviously in both GES-1/siCREB1 and GES-1/CREB1 (Fig. 5A). The same trend of MBNL1 was observed in BGC823 cells (Fig. S5C and S5D). IHC study showed that the expression of MBNL1 was positively correlated with CREB1 in tumor samples (n = 56, *r* = 0.419, *P* = 0.01), and the correlation between CREB1 and MBNL1 was insignificant in normal gastric samples (n = 52, *r* = 0.153, *P* = 0.276) (Fig. 5B, C).

Taken together, western blot and IHC results confirmed the effect of differential regulation of TCEAL2 and MBNL1 by CREB1 at the protein level, supporting the hypothesis on the dysfunctional mechanism of CREB1 in gastric carcinogenesis.

## Discussion

Cancer is one of major health challenges for humanity, due to its complex molecular characteristics, tumor microenvironment, immune privilege, metastatic capacity and so on (Hanahan, 2022; Isik et al. 2020, 2021). Gastric carcinogenesis is a multistep process with genomic changes in genes controlling cell growth and differentiation, followed by the dysregulation of cell signaling transduction, which leads to abnormal expression of a large number of genes, and eventually over-activation of cell proliferation. In the field of transcriptomics, DCEA has proved to be an effective strategy to explore gene interconnection changes under varying conditions (Cao et al. 2015; Dai et al. 2018; de la Fuente, 2010; Li et al. 2017a; Yu et al. 2011). DCEA looks at changes in gene co-expression patterns, and thus provides clues to the disrupted regulatory relationships or dysfunctional regulations specific to interested phenotype (Liu et al. 2010). Our previous work has integrated this strategy and reverse-forward integrated modeling method to obtain three stage-specific (normal, adenoma and cancer) differential regulation-enriched networks based on TCGA-STAD dataset, leading to 36 DRGs for normal to adenoma transition and 56 DRGs for adenoma to cancer transition, out of which more than 50% have been reported to be GC related (Cao et al. 2015). TFs as central regulators of gene expression are involved in the initiation, maintenance and progression of tumor. Mutations or aberrant expression of oncogenic TFs in tumor have been frequently demonstrated, such as SOX2 in esophageal squamous cancer (Watanabe et al. 2014), NKX2-1 in lung adenocarcinoma (Mollaoglu et al. 2018) and AR in prostate cancer (Culig and Santer 2014), however, their dysfunctional transcriptional regulations have been seldom studied, especially in gastric carcinogenesis.

In order to find out the dysregulation mechanisms underlying gastric carcinogenesis, we focused on GC-related TFs and their surrounding top ranked DRLs. During disease progression, those abnormal regulation relationships, which might be disrupted expression correlation or emerging correlations between TFs and targets, could be captured by our integrative strategy including DCEA, GRN modeling and DRA. Based on dataset GSE54129, involving 111 gastric cancer and 21 normal mucosa samples, we identified four TFs including CREB1, BPTF, GATA6 and CEBPA with high DR value among top 1% DRGs. In order to investigate the dynamic regulation changes of TFs from normal to cancer, we focused on the oncogenic TF—CREB1 and identified two differentially regulated target genes, TCAEL2 and MBNL1, experimentally validating the differential dependence of target’s mRNA expression on the expression level of CREB1 between normal and cancer.

TCEAL2 has been recognized as an important nuclear target for intracellular signal transduction. Several studies have observed that TCEAL2 was overexpressed in various tumors including meningioma and ovarian carcinoma, and it was closely related to poor clinical outcome (Kim et al. 2010; Stuart et al. 2011). Though TCEAL2 is not a known GC-related gene, it was reported to be a potential immunotherapeutic target in SCLC and could be involved in promoting proliferation and inhabiting apoptosis of cancer cells (Taguchi et al. 2014). The overexpression of TCEAL2 in cancer condition was observed in our GSE54129 dataset (Additional file 6: Fig. S6A), meanwhile the loss of negative regulation of TCEAL2 by CREB1 in cancer was inferred, which may facilitate proliferation and inhibit apoptosis and thus promotes carcinogenesis.

MBNL1 was one of pre-mRNA alternative splicing factors, and affected many steps of RNA maturation and expression (Han et al. 2013). A recent study suggested MBNL1 could suppress breast cancer metastatic colonization and stabilize metastasis suppressor transcripts (Fish et al. 2016), while another study revealed that MBNL1 was a cancer-related splicing regulator which acted as a splicing repressor in Dicer1 processing, resulting in colorectal carcinogenesis (Tang et al. 2015). The overexpression of MBNL1 was observed in our present dataset, in accordance with the emerging positive regulation of MBNL1 by CREB1 in cancer (Additional file 6: Fig. S6B). We therefore proposed that the positive regulation of MBNL1 by CREB1 might lead to the overexpression of MBNL1 that promotes GC progression via Dicer1 processing.

Gene correlation analysis and survival analysis of CREB1, TCEAL2 and MBNL1 on TCGA-STAD dataset were also performed. We observed no correlation between CREB1 and TCEAL2 (Spearman correlation coefficient is 0.042, p = 0.4) and significant correlation between CREB1 and MBNL1 (Spearman correlation coefficient is 0.64, p < 0.001) (Additional file 7: Fig. S7A, B), which is consistent with the scenario based on GSE54129. Kaplan–Meier survival analyses showed that the two groups classified by CREB1 expression level has no significant difference (HR = 1.2, pHR = 0.21; Additional file 7: Fig. S7C) on the overall survival (OS). However, when adopting CREB1, TCEAL2 and MBNL1 as a 3Sginature, the High-3Signatures group displayed a significantly poor OS compared with the Low-3Signatures group (HR = 1.4, pHR = 0.033; Additional file 7: Fig. S7D). Therefore, the main conclusions of our manuscript were basically validated on the TCGA-STAD dataset.

Through mutual verification of computational analysis, clinical pathology and biological experiments, we have obtained reliable and consistent results to support our hypothesis on the dysfunctional mechanism of CREB1 during gastric cancer progression. Besides potential CREB1/TCEAL2-MBNL1 signaling, our DCEA-GRN-DRA framework has actually provided several other insightful hints on dysfunctional regulation mechanisms underlying carcinogenesis although we have not yet discover the causal factors of dysregulation events in the present work. A plausible speculation is that under the repeated stimulation of various external factors in the gastric environment, the metabolism of gastric epithelial cells and stromal cells changes, which may lead to the mutation of TF DNA binding domain, the phosphorylation and histone modification of TFs, or recruitment of different co-factors, resulting in the alteration of regulation pattern of a specific TF across different stages. The detailed mechanisms and their roles in GC progression are obviously worthy of further investigation. The lack of effective treatments for advanced cancer is a major challenge in clinical oncology therapeutics. Considering that the transcriptional activities of TFs are hard to be inhibited directly, the targets and pathways they regulate might be more tractable for drug development.

## Conclusions

In summary, by combining differential networking information and molecular cell experiments verification, we generated and verified the testable hypotheses on the dysregulation mechanisms of GC around the crucial TFs and their top ranked DRLs, and proposed a CREB1/TCEAL2-MBNL1 signaling model, where TCEAL2 and MBNL1 were proved to be differentially regulated by CREB1 during tumorigenesis of gastric cancer. We therefore proposed TCEAL2 and MBNL1 as potential therapeutic targets for gastric cancer. Furthermore, our DCEA-GRN-DRA data mining framework has been proved to have the potential to generate new insights into the dysfunctional regulation mechanisms underlying carcinogenesis.

## Supplementary Information

**Additional file 1: Figure S1. Quality assessment of dataset (GSE54129). A.** Distribution of log2 transformed expression level of genes in different samples. **B.** Density distribution of expression level of 132 samples. **C.** The correlations among 132 samples. The number ID 1 to 21 represent normal samples, others are cancer samples. **D.** Clustering based on gene expression of GSE54129 dataset. This clustering shows distinct groups of samples. All of cancer samples are clustered together.

**Additional file 2: Figure S2. The proposed dysregulation mechanisms around BPTF. A.** The expression level of BPTF in GSE54129 dataset. **B.** BPTF is a TF, and the other nodes are its targets. Links in red, blue and grey represent positive, negative and absent relationships calculated with dataset GSE54129. Numbers on the links indicate the regulation efficacies. **C.** The proposed mechanism by which BPTF induces GC. Links in red, blue and grey still represent positive, negative and absent regulation relationships at normal and cancer stages calculated with dataset GES54129. Links in black are gene–gene interconnections obtained from literature. The color of gene symbol, red or blue, represents up- or down-expression in stage transition according to dataset GES54129. The box indicates biological processes, with red color referring to activation and blue color referring to inhibition. * means significant *P*-value < 0.05, two-sided Student’s *t*-test.

**Additional file 3: Figure S3. The proposed dysregulation mechanisms around GATA6. A.** The expression level of GATA6 in GSE54129, * means significant *P*-value < 0.05. **B.** GATA6 is a TF, and the other nodes are its targets. **C.** The proposed mechanism by which GATA6 induces GC. Links in red, blue and grey still represent positive, negative and absent regulation relationships at normal and cancer stages calculated with dataset GES54129. Links in black are gene–gene interconnections obtained from literature. The color of gene symbol, red or blue, represents up- or down-expression in stage transition according to dataset GES54129.

**Additional file 4: Figure S4. The proposed dysregulation mechanisms around CEBPA. A.** The expression level of CEBPA in GSE54129, * means significant *P*-value < 0.05. **B.** CEBPA is a TF, and the other nodes are its targets. Links in red, blue and grey represent positive, negative and absent relationships calculated with dataset GSE54129. Numbers on the links indicate the regulation efficacies. **C.** The proposed mechanism by which CEBPA induces GC.

**Additional file 5: Figure S5.** The mRNA and protein levels of MBNL1 and TCEAL2 were measured by qRT-PCR and western blot in BGC823 cells disturbed by CREB1 overexpression or knockdown. * means significant *P*-value < 0.05, two-sided Student’s *t*-test.

**Additional file 6: Figure S6.** TCEAL2 (**A**) and MBNL1 (**B**) expression levels in GSE54129 dataset. * means significant *P*-value < 0.05, NS means no significant, two-sided Student’s *t*-test.

**Additional file 7: Figure S7.** The expression relationship of TCEAL2 (**A**) and MBNL1 (**B**) with CREB1 on the TCGA-STAD dataset. **C.** The overall survival curves of patients with different groups by CREB1's expression level on the TCGA-STAD dataset. **D.** The overall survival curves of patients with High- or Low-3Sginature (CREB1—TCEAL2—MBNL1) on the TCGA-STAD dataset.

**Additional file 8: Table S1. **Primers pairs for in vitro assays.

**Additional file 9: Table S2.** The DRG list in a descending order by DR value between normal and cancer.

## Data Availability

All data are available at the National Center for Biotechnology Information (NCBI) Gene Expression Omnibus (GEO) (http://www.ncbi.nlm.nih.gov/geo) database (GSE54129).

## Abbreviations

Bet: Betweenness
CC: Clustering coefficient
Cls: Closeness
DCEA: Differential co-expression analysis
DCG: Differentially co-expressed gene
DCL: Differentially co-expressed gene link
DEG: Differentially expressed gene
DR: Differential regulation
DRA: Differential regulation analysis
DRL: Differentially regulated link
GC: Gastric cancer
GRN: Gene regulatory network
In-Deg: In-degree
Out-Deg: Out-degree
TDR: Diversity of a regulator
TF: Transcription factor
TCGA: The Cancer Genome Atlas
STAD: Stomach adenocarcinoma
OS: Overall survival
