# Supplementary figures and images for: The dynamic alteration of transcriptional regulation by crucial TFs during tumorigenesis of gastric cancer
Source: Mol Med. 2022 Apr 14;28:41. doi: 10.1186/s10020-022-00468-7 (PMC9008954; doi:10.1186/s10020-022-00468-7)

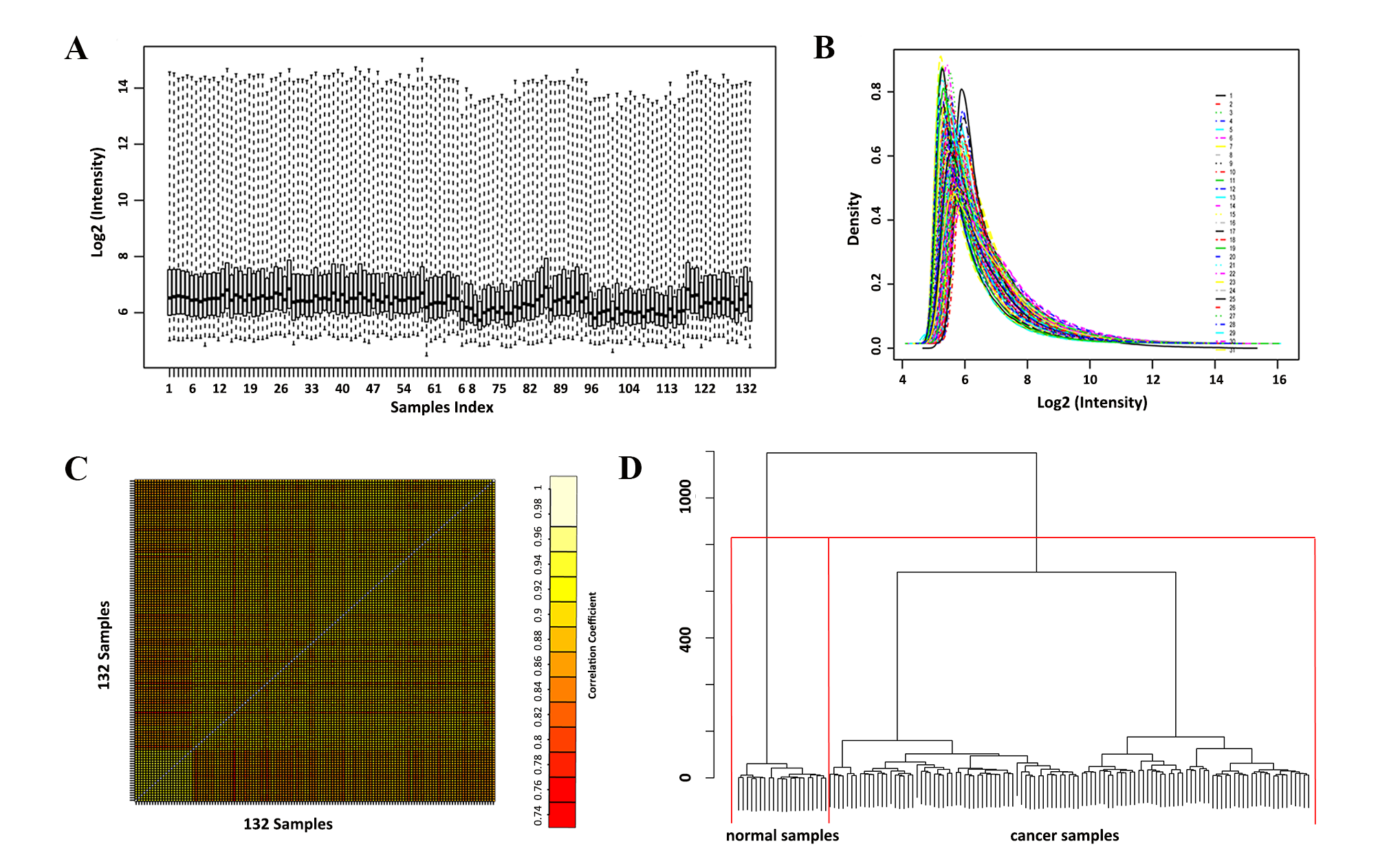

Supplement: Supplementary file 1 — Additional file 1: Figure S1. Quality assessment of dataset (GSE54129). A. Distribution of log2 transformed expression level of genes in different samples. B. Density distribution of expression level of 132 samples. C. The correlations among 132 samples. The number ID 1 to 21 represent normal samples, others are cancer samples. D. Clustering based on gene expression of GSE54129 dataset. This clustering shows distinct groups of samples. All of cancer samples are clustered together. [file 10020_2022_468_MOESM1_ESM.tif]

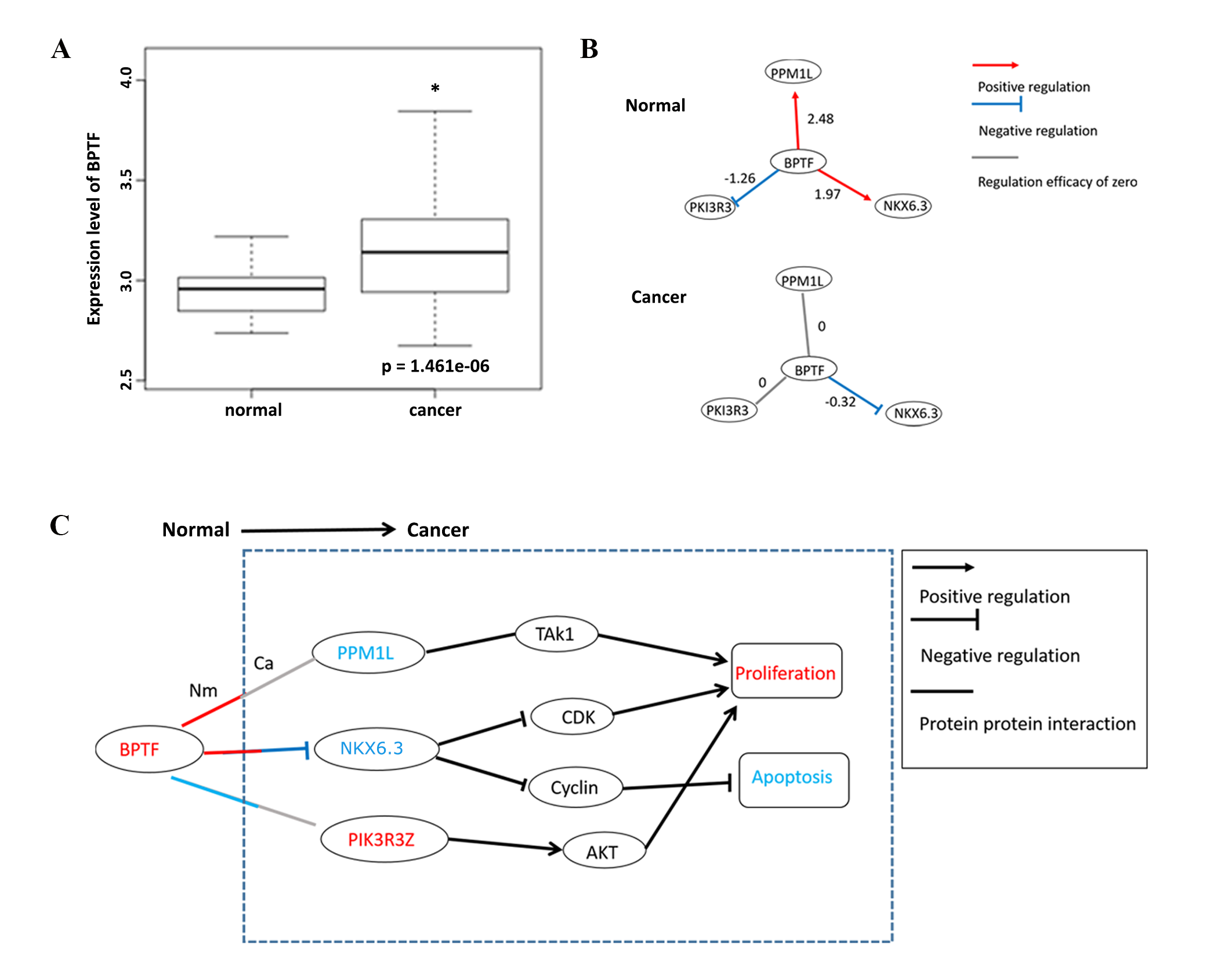

Supplement: Supplementary file 2 — Additional file 2: Figure S2. The proposed dysregulation mechanisms around BPTF. A. The expression level of BPTF in GSE54129 dataset. B. BPTF is a TF, and the other nodes are its targets. Links in red, blue and grey represent positive, negative and absent relationships calculated with dataset GSE54129. Numbers on the links indicate the regulation efficacies. C. The proposed mechanism by which BPTF induces GC. Links in red, blue and grey still represent positive, negative and absent regulation relationships at normal and cancer stages calculated with dataset GES54129. Links in black are gene–gene interconnections obtained from literature. The color of gene symbol, red or blue, represents up- or down-expression in stage transition according to dataset GES54129. The box indicates biological processes, with red color referring to activation and blue color referring to inhibition. * means significant P-value < 0.05, two-sided Student’s t-test. [file 10020_2022_468_MOESM2_ESM.tif]

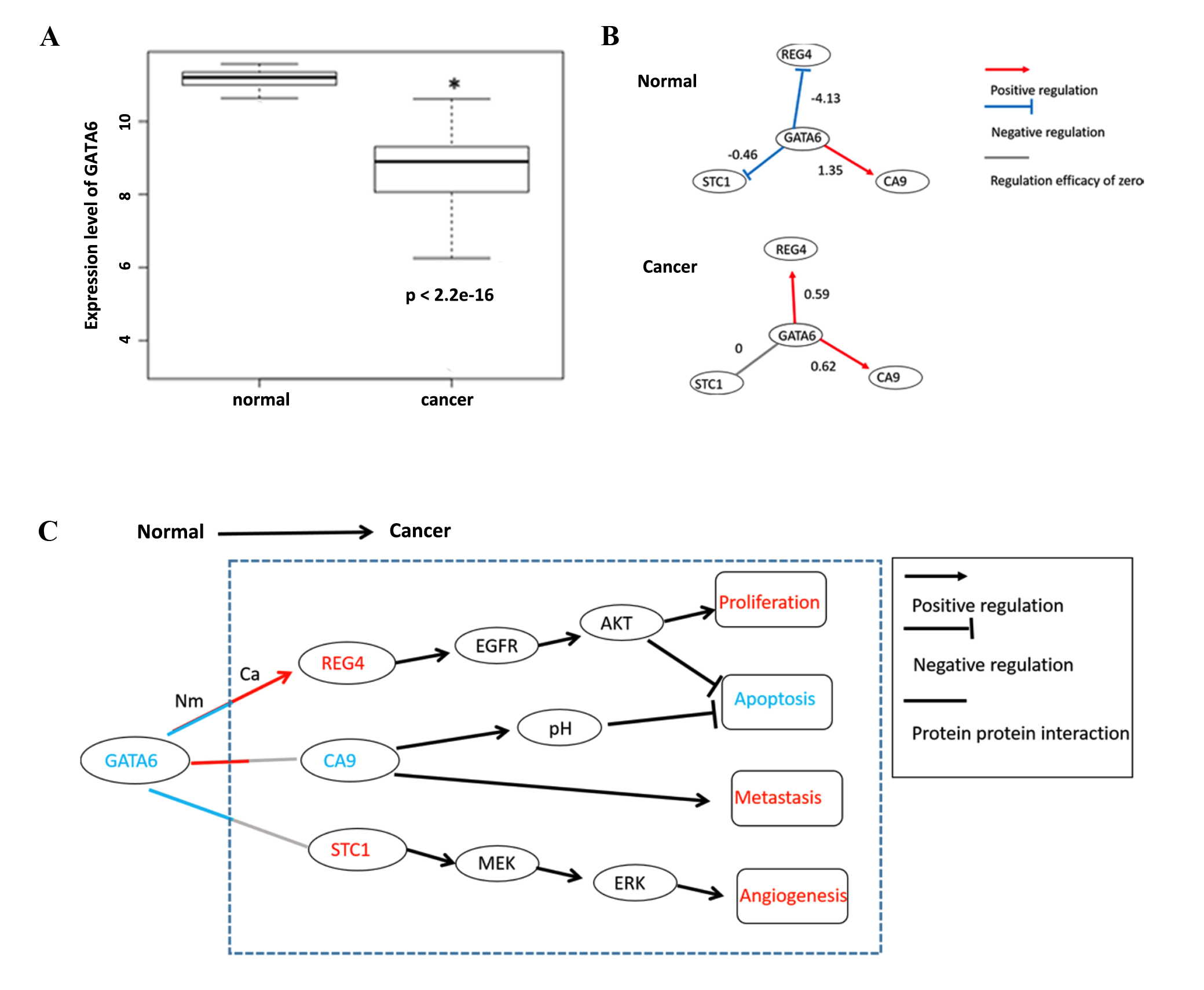

Supplement: Supplementary file 3 — Additional file 3: Figure S3. The proposed dysregulation mechanisms around GATA6. A. The expression level of GATA6 in GSE54129, * means significant P-value < 0.05. B. GATA6 is a TF, and the other nodes are its targets. C. The proposed mechanism by which GATA6 induces GC. Links in red, blue and grey still represent positive, negative and absent regulation relationships at normal and cancer stages calculated with dataset GES54129. Links in black are gene–gene interconnections obtained from literature. The color of gene symbol, red or blue, represents up- or down-expression in stage transition according to dataset GES54129. [file 10020_2022_468_MOESM3_ESM.tif]

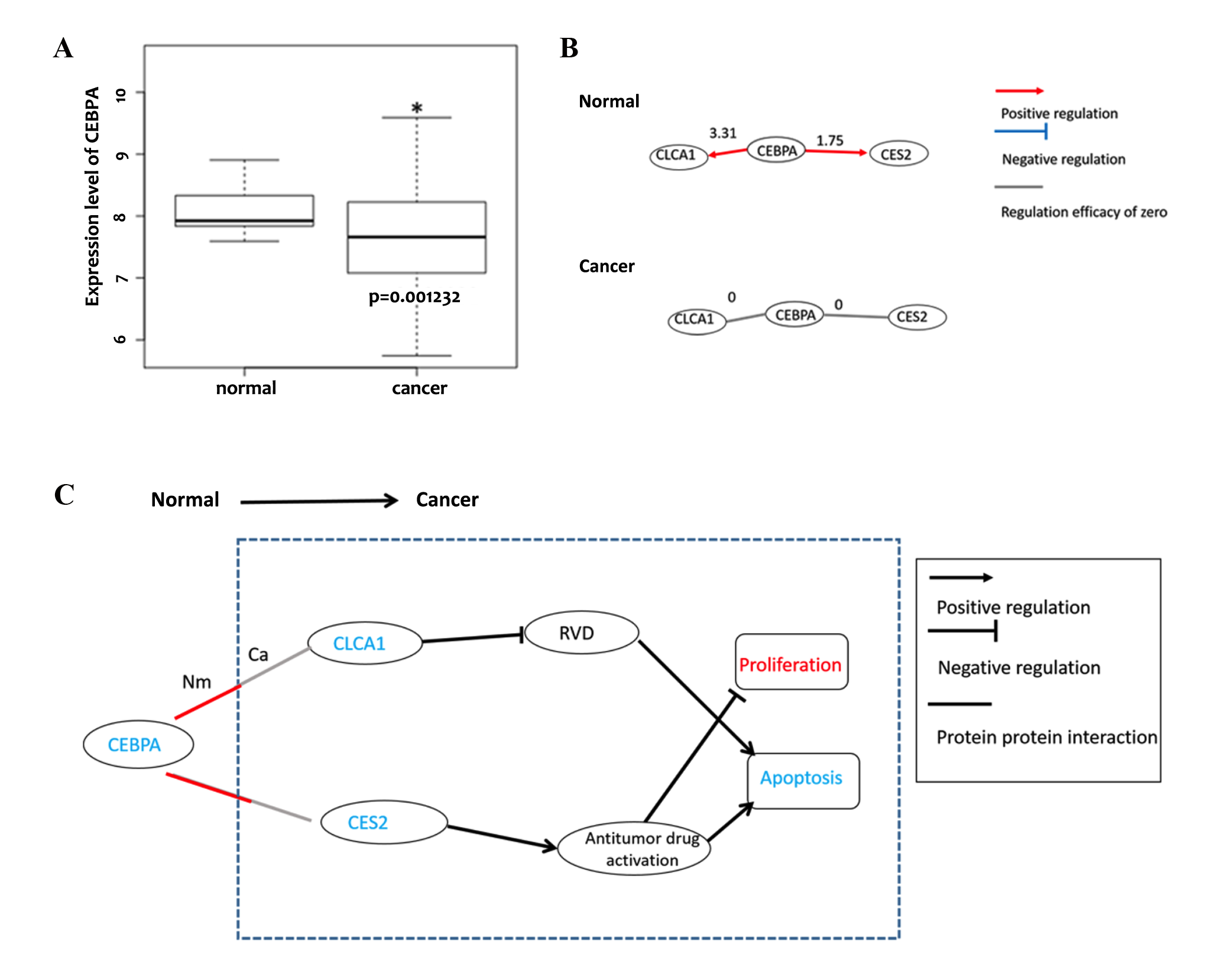

Supplement: Supplementary file 4 — Additional file 4: Figure S4. The proposed dysregulation mechanisms around CEBPA. A. The expression level of CEBPA in GSE54129, * means significant P-value < 0.05. B. CEBPA is a TF, and the other nodes are its targets. Links in red, blue and grey represent positive, negative and absent relationships calculated with dataset GSE54129. Numbers on the links indicate the regulation efficacies. C. The proposed mechanism by which CEBPA induces GC. [file 10020_2022_468_MOESM4_ESM.tif]

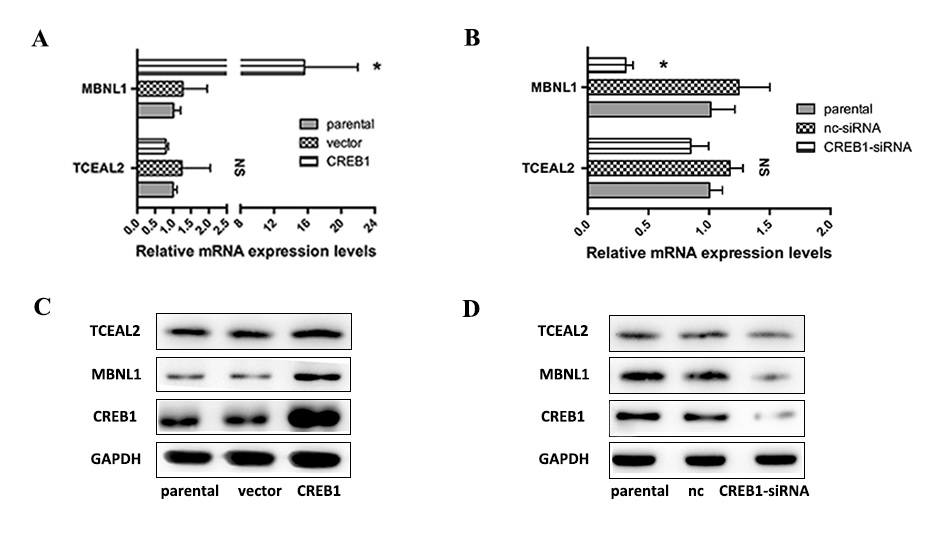

Supplement: Supplementary file 5 — Additional file 5: Figure S5. The mRNA and protein levels of MBNL1 and TCEAL2 were measured by qRT-PCR and western blot in BGC823 cells disturbed by CREB1 overexpression or knockdown. * means significant P-value < 0.05, two-sided Student’s t-test. [file 10020_2022_468_MOESM5_ESM.tif]

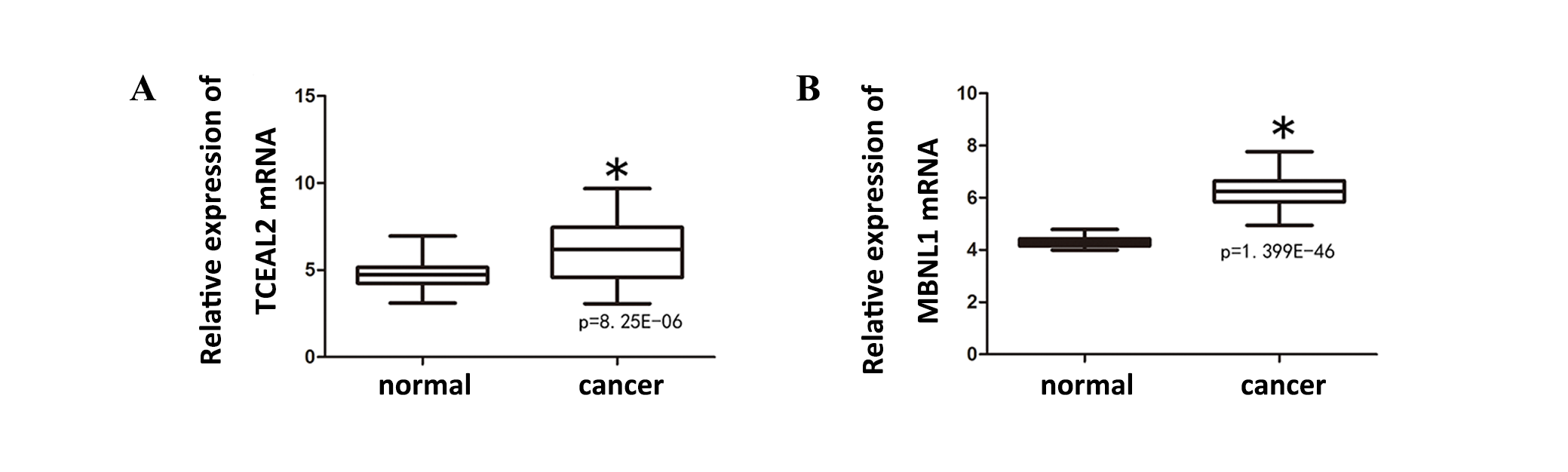

Supplement: Supplementary file 6 — Additional file 6: Figure S6. TCEAL2 (A) and MBNL1 (B) expression levels in GSE54129 dataset. * means significant P-value < 0.05, NS means no significant, two-sided Student’s t-test. [file 10020_2022_468_MOESM6_ESM.tif]

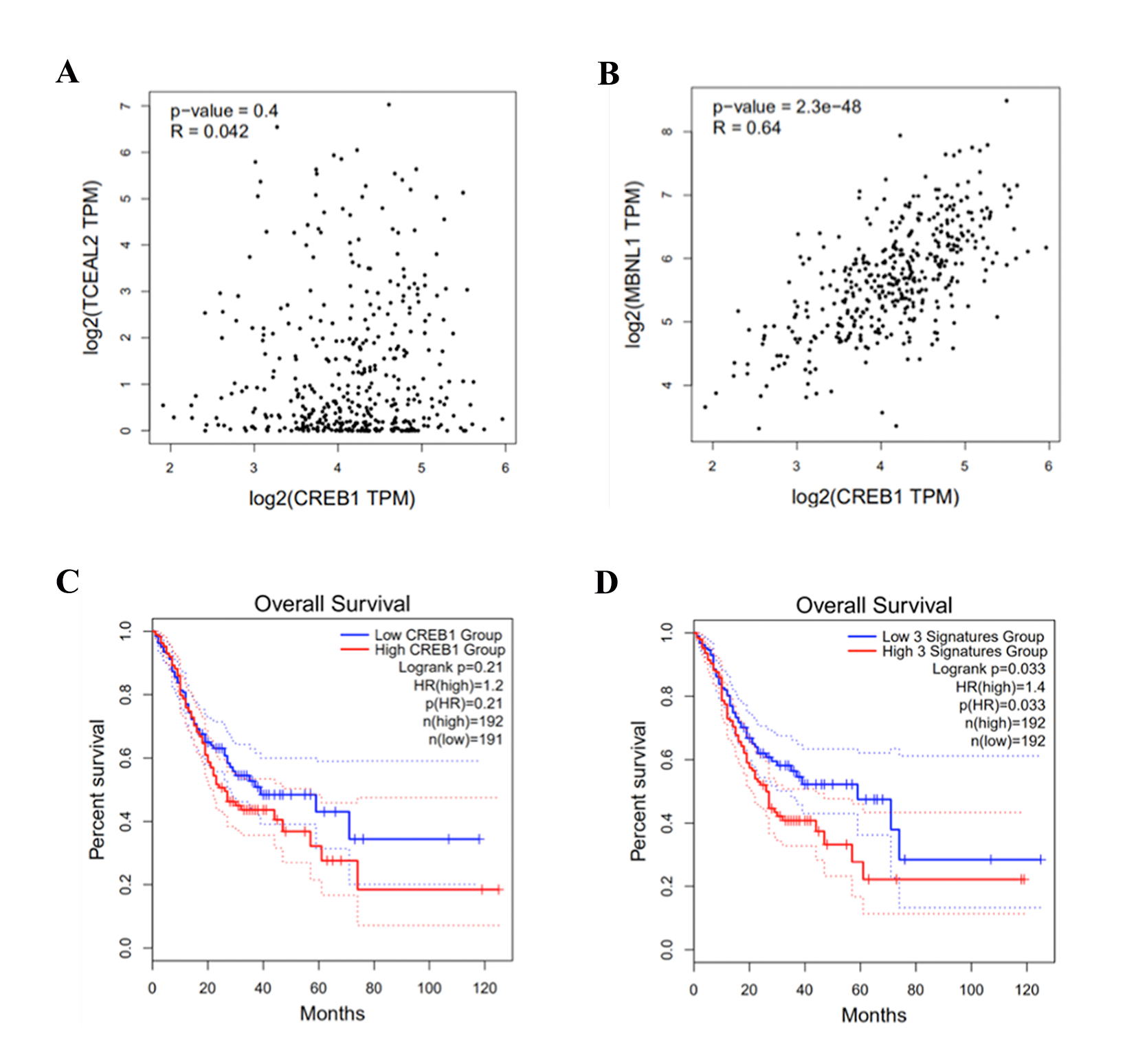

Supplement: Supplementary file 7 — Additional file 7: Figure S7. The expression relationship of TCEAL2 (A) and MBNL1 (B) with CREB1 on the TCGA-STAD dataset. C. The overall survival curves of patients with different groups by CREB1's expression level on the TCGA-STAD dataset. D. The overall survival curves of patients with High- or Low-3Sginature (CREB1—TCEAL2—MBNL1) on the TCGA-STAD dataset. [file 10020_2022_468_MOESM7_ESM.tif]
