# Supplementary material for: The dynamic alteration of transcriptional regulation by crucial TFs during tumorigenesis of gastric cancer
Source: Mol Med. 2022 Apr 14;28:41. doi: 10.1186/s10020-022-00468-7 (PMC9008954; doi:10.1186/s10020-022-00468-7)
Supplement: Supplementary file 8 — Additional file 8: Table S1. Primers pairs for in vitro assays. [file 10020_2022_468_MOESM8_ESM.docx]

**Table S1. Primers pairs for in vitro assays**

| **Primer Name** | **Sequence (5' -> 3')** |
| --- | --- |
| GAPDH Forward | TTGGCATCGTTGAGGGTCT |
| GAPDH Reverse | CAGTGGGAACACGGAAAGC |
| CREB1 Forward | CCACTGTAACGGTGCCAACT |
| CREB1 Reverse | GCTGCATTGGTCATGGTTAATGT |
| TRIM15 Forward | TCCCTGAAGGTGGTCCATGAG |
| TRIM15 Reverse | CAGGATCTTGCCCGAGGATT |
| TCEAL2 Forward | TCGTTCTCGCCCGCAATTTAG |
| TCEAL2 Reverse | GATAAGGACGGCTCCGTTTTG |
| NHERF1 Forward | GGCTGGCAACGAAAATGAGC |
| NHERF1 Reverse | TGTCGCTGTGCAGGTTGAAG |
| RBPMS2 Forward | CTGAACGGTATTCGCTTTGATCC |
| RBPMS2 Reverse | GTCCCGTGCGATGAAGTGT |
| FERMT2 Forward | CAGACACCCCGAAGAACTTTC |
| FERMT2 Reverse | GCCCCTCTAATTCAAGTGCCT |
| FAM20C Forward | GGCACAATGCGGAGATTGC |
| FAM20C Reverse | CAGAGCTTCTTGTCCCGTGT |
| MBNL1 Forward | GCTGTTAGTGTCACACCAATTCG |
| MBNL1 Reverse | AGGCGATTACTCGTCCATTTTC |
